# Rheological Flow Behavior of Six Gelling Agents and Their Relevance for In Vitro Culture Performance of Medicinal Plants

**DOI:** 10.3390/gels12020163

**Published:** 2026-02-12

**Authors:** Doina Clapa, Monica Hârţa, Bernadette-Emőke Teleky, Ana-Maria Radomir, Adrian George Peticilă, Dorin Ioan Sumedrea

**Affiliations:** 1 Faculty of Horticulture and Business in Rural Development, University of Agricultural Sciences and Veterinary Medicine of Cluj-Napoca, Mănăştur St. 3-5, 400372 Cluj-Napoca, Romania; doina.clapa@usamvcluj.ro; 2 Institute of Life Sciences, University of Agricultural Sciences and Veterinary Medicine, Calea Mănăştur 3-5, 400372 Cluj-Napoca, Romania; bernadette.teleky@usamvcluj.ro; 3 National Research and Development Institute for Biotechnology in Horticulture Ștefănești-Argeș, 37 Bucharest-Pitești Road, 117715 Ștefănești, Romania; 4 Faculty of Horticulture, University of Agronomic Sciences and Veterinary Medicine of Bucharest, 59 Mărăşti Blvd, District 1, 011464 Bucharest, Romania; apeticila@gmail.com; 5 The Academy of Agricultural and Forestry Sciences, 61 Mărăşti Blvd, District 1, 011464 Bucharest, Romania; dsumedrea@yahoo.com

**Keywords:** gelrite, low-shear viscosity, micropropagation, rheology, shear-thinning behavior

## Abstract

Gelling agents are widely used to solidify plant tissue culture media, yet differences among commercial products may influence the medium’s physical properties and in vitro development of explants. The aim of this study was to characterize the rheological behavior of six gelling agents (Daishin agar, Gelcarin, Gelrite, Microagar, Phytoagar, and Plant agar) and to examine it in parallel with in vitro performance in *Hypericum perforatum*, *Mentha* × *piperita*, and *Stevia rebaudiana*. Rheological measurements were performed under steady shear by recording apparent viscosity and shear stress across 5–300 s^−1^. Daishin agar showed the highest apparent viscosity (49,028.95 ± 128 mPa·s), whereas Gelrite exhibited the lowest viscosity (7826.75 ± 98 mPa·s). Plant responses were evaluated after four weeks on PGR-free Driver and Kuniyuki Walnut (DKW) medium by assessing shoot growth, rooting parameters, and shoot water content. In *H. perforatum*, the longest shoots were obtained on Gelrite (3.92 ± 0.34 cm), accompanied by the highest rooting percentage (95%). In *M.* × *piperita*, Gelcarin produced the longest shoots (8.20 ± 0.55 cm) and the highest number of roots per explant (9.75). In *S. rebaudiana*, Gelcarin promoted superior root elongation (2.86 ± 0.16 cm) and enhanced shoot growth, while Plant agar also supported favorable shoot development. Shoot water content ranged between 74% and 90%, depending on species and gelling agent. These findings highlight the practical relevance of considering low-shear rheological properties when comparing gelling agents for improving the consistency of in vitro culture media.

## 1. Introduction

In conventional in vitro culture systems, plant growth occurs on solid or semi-solid media, where the gelling agent plays a key role in ensuring physical stability and regulating water availability at the explant–medium interface [1,2]. Variations in gelling agents can significantly influence morphogenic responses such as shoot development, rooting, and callus formation [3,4]. The gel matrix can contribute to shaping the microenvironment of cultured explants by affecting the mobility of water and dissolved nutrients within the medium. Since calcium availability and transport have been repeatedly associated with shoot tip necrosis and reduced culture performance, optimizing gelled media may indirectly support improved nutrient balance and plantlet quality during in vitro culture [5]. Recent studies have highlighted that gelling agent types and concentrations act as critical factors or ‘bottlenecks’ in in vitro systems, directly influencing nutrient availability and mass transfer at the explant–medium interface [6]. Commercial products used for medium gelation differ in purity, gel strength, and water diffusion properties, which may result in distinct growth and regeneration responses in vitro [7,8]. Moreover, agar-based products are not chemically uniform, and their physical behavior depends on factors such as the red algae source, extraction process, and concentration applied in the culture medium [9,10]. Since conventional in vitro cultures are essentially static systems, differences in low-shear viscosity may be especially relevant for explant support, hydration balance, and mass transfer at the gel surface. Moreover, gelling agent selection is often based on routine use or availability rather than on quantified rheological performance, which may contribute to variability and reproducibility issues across laboratories.

Rheological characterization provides a quantitative approach for describing the flow behavior of gelling systems relevant to plant tissue culture. Parameters such as apparent viscosity and shear stress under controlled shear conditions can be used to compare gel performance and support the selection of suitable gelling agents for reproducible in vitro culture media [11,12,13,14]. Studies show that the physical and mechanical properties of gelling agents affect in vitro plant tissue responses like callus growth and shoot development. However, most research has focused on single species or limited systems, rarely addressing low-shear rheological behavior relevant to static in vitro conditions. Also, studies systematically linking the rheological properties of commercially available gelling agents with in vitro growth outcomes in plants, including medicinal species, remain limited.

Medicinal plants are cultivated extensively as sources of bioactive compounds for therapeutic applications, functional foods, cosmetics, and phytopharmaceutical products [15,16,17,18]. Among them, *Hypericum perforatum* L., *Mentha* × *piperita* L., and *Stevia rebaudiana* Bertoni are widely valued due to their phytochemical profiles and industrial relevance [19,20,21]. The development of rapid and standardized in vitro protocols is essential to ensure the large-scale production of uniform and pathogen-free plant material, supporting both conservation strategies and commercial supply [22,23,24].

Recent advances in the in vitro culture of *H. perforatum* have demonstrated the feasibility of efficient micropropagation, regeneration, and biomass production using a wide range of explants and culture systems. A comprehensive recent review highlighted that MS-based media are most commonly used for shoot proliferation and regeneration, typically solidified with agar, while alternative gelling agents such as Gelrite and Phytagel have also been employed in specific protocols, particularly for shoot cultures and adventitious root induction. These studies emphasize that, in addition to basal medium composition and plant growth regulators, the physical properties of the solidification system may influence shoot quality, rooting efficiency, and tissue hydration in vitro [25]. For *M*. × *piperita*, recent in vitro studies have reported efficient micropropagation from nodal explants, characterized by high shoot multiplication rates and spontaneous rooting on MS-based media under optimized culture conditions [26]. It has been shown that in vitro growth and rooting responses in Mentha are sensitive to medium composition and culture conditions, affecting shoot elongation and overall plantlet quality [27,28,29]. Likewise, several recent reports have shown that shoot proliferation, elongation, rooting efficiency, and tissue hydration in *S. rebaudiana* are influenced by the physical properties of the culture system, with studies comparing agar- and Gelrite-solidified media reporting gelling-agent-dependent differences in in vitro performance and plantlet quality [30,31,32,33,34].

Despite their widespread use, gelling agents are often selected in plant tissue culture based on availability or tradition rather than on defined physical or rheological criteria. Different commercial products can differ substantially in gel strength, viscosity, and water-holding capacity, which may alter nutrient diffusion, mechanical support, and water availability at the explant–medium interface. These factors can directly affect shoot elongation, rooting efficiency, and tissue hydration, potentially leading to inconsistent in vitro performance or physiological disorders. However, most previous studies have evaluated gelling agents primarily based on biological outcomes, without systematically relating plant responses to measurable rheological properties of the gel matrix. As a result, the lack of clear selection criteria for gelling agents remains a practical limitation in the optimization of in vitro culture media.

Therefore, this study aimed to evaluate whether rheological flow properties of six commercially available gelling agents (Daishin agar, Gelcarin, Gelrite, Microagar, Phytoagar, and Plant agar) can serve as a practical criterion for their selection in in vitro culture, by integrating rheological characterization with biological performance in *H. perforatum*, *M.* × *piperita*, and *S. rebaudiana*. Shoot and root development, rooting percentage, shoot water content, and gel flow properties were assessed under standardized conditions to relate gel mechanics and hydration properties to in vitro culture responses.

## 2. Results

### 2.1. Flow Behavior and Apparent Viscosity of the Gelling Agents

**Figure 1:**
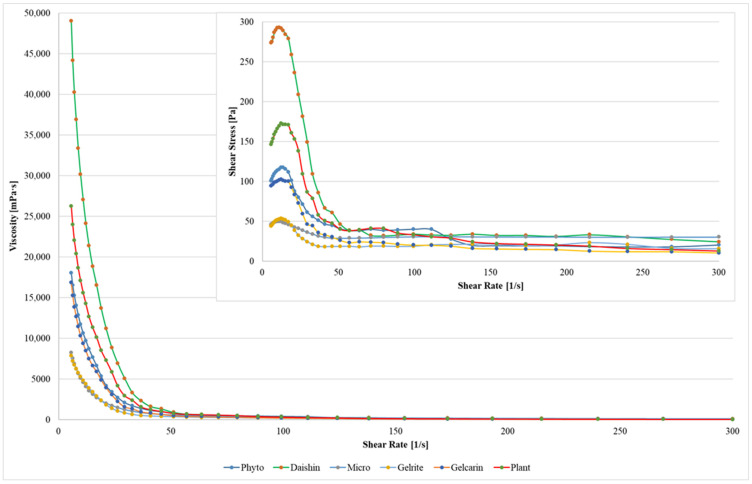
Apparent viscosity (mPa·s) and shear stress (Pa) as a function of shear rate (5–300 s^−1^) for six gelling agents used for in vitro culture media (Daishin agar, Gelcarin, Gelrite, Microagar, Phytoagar, and Plant agar). Curves represent mean values obtained from steady shear measurements performed in duplicate at room temperature.

Figure 1 presents the flow behavior of the six gelling agents evaluated under steady shear conditions, showing apparent viscosity and shear stress as a function of shear rate (5–300 s^−1^). The results highlight marked differences among the tested gel systems, particularly at low shear rates, where the initial gel structure is expected to have the strongest influence on resistance to flow.

At low shear rates, Daishin agar exhibited the highest apparent viscosity (49,028.95 ± 128 mPa·s), followed by Plant agar (26,232.6 ± 112 mPa·s) and Phytoagar (18,053.2 ± 108 mPa·s). In contrast, Gelrite displayed the lowest viscosity (7826.75 ± 98 mPa·s), indicating a substantial variation in the flow resistance of the tested gelling agents. As the shear rate increased, the viscosity decreased sharply for all samples, resulting in a progressive convergence of the curves at higher shear rates.

### 2.2. In Vitro Culture Performance on Different Gelling Agents

**Figure 2:**
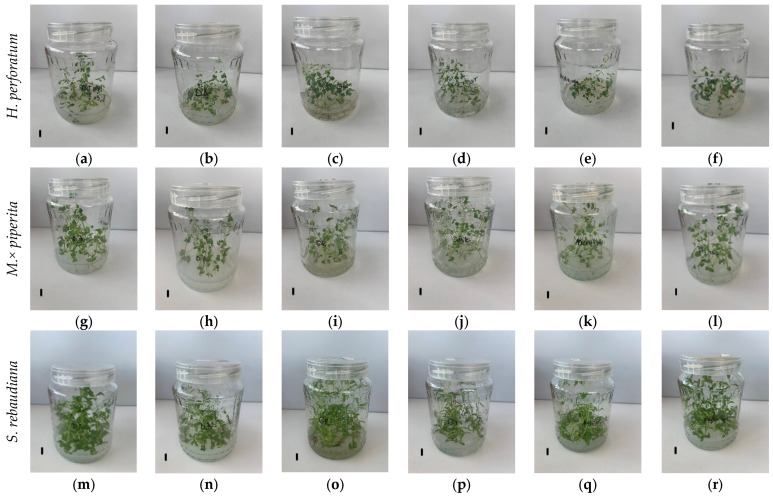
Representative in vitro culture appearance of *H. perforatum* (**a**–**f**), *M.* × *piperita* (**g**–**l**), and *S. rebaudiana* (**m**–**s**) grown on PGR-free DKW basal medium solidified with different gelling agents after four weeks of culture: Daishin agar (**a**,**g**,**m**), Gelcarin (**b**,**h**,**n**), Gelrite (**c**,**i**,**o**), Microagar (**d**,**j**,**p**), Phytoagar (**e**,**k**,**q**), and Plant agar (**f**,**l**,**r**). A scale bar (1 cm) is shown in each panel. All images were acquired using identical culture vessels (720 mL jars; 13.5 cm × 9 cm).

**Figure 3:**
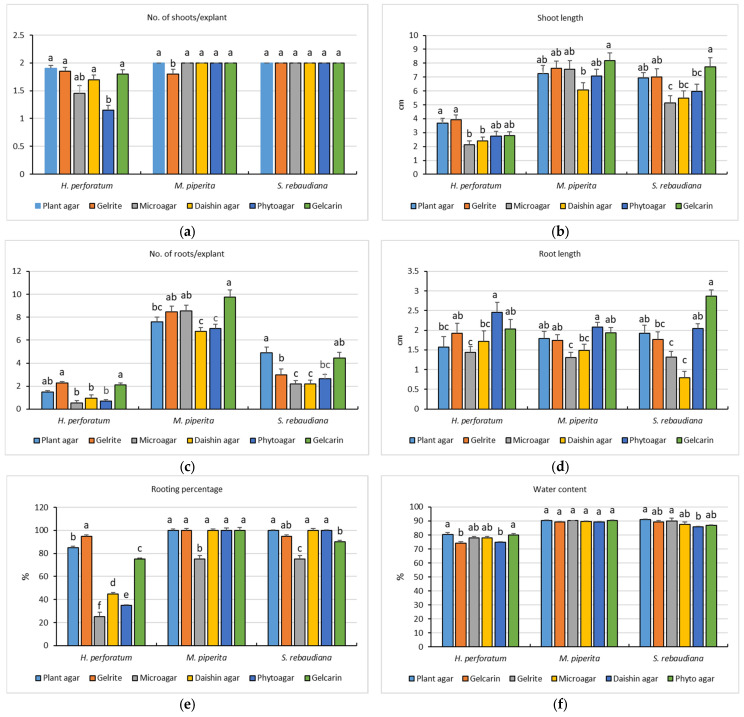
In vitro culture performance of *H. perforatum*, *M.* × *piperita*, and *S. rebaudiana* grown on PGR-free DKW basal medium solidified with different gelling agents after four weeks: (**a**) number of shoots/explant, (**b**) shoot length (cm), (**c**) number of roots/explant, (**d**) root length (cm), (**e**) rooting percentage (%), and (**f**) shoot water content (%). Bars represent mean ± SE (*n* = 3 culture vessels, with 10 explants per vessel). Different letters indicate significant differences among gelling agents within each species (Tukey’s HSD, *p* < 0.05).

Based on the marked differences in apparent viscosity and shear stress responses observed among the tested gelling agents, their functional performance was further evaluated in vitro. The representative in vitro culture appearance is shown in Figure 2, while quantitative comparisons of shoot growth, rooting response, and shoot water content are presented in Figure 3. Results are presented separately for each species, which were treated as distinct biological units. Accordingly, differences among gelling agents were evaluated within each species, without direct statistical comparisons between species.

#### 2.2.1. Shoot Growth Response

Shoot number per explant (Figure 3a) remained close to 2 in most treatments, which is expected under the applied culture conditions (DKW basal medium without plant growth regulators). In *M.* × *piperita* and *S. rebaudiana*, shoot numbers were approximately 2.0 shoots/explant across all gelling agents, and no statistically significant differences were detected among treatments (Tukey’s HSD, *p* > 0.05).

In *H. perforatum*, slight but statistically significant variation was recorded among gelling agents (Tukey’s HSD, *p* < 0.05), with Plant agar (1.90 ± 0.05 shoots/explant) and Gelrite (1.85 ± 0.06 shoots/explant) showing the highest values, while Phytoagar resulted in the lowest shoot number (1.15 ± 0.09 shoots/explant).

In contrast, shoot length was clearly affected by the type of gelling agent, and the response differed among species (Figure 3b). In *H. perforatum*, shoot length ranged from 2.14 ± 0.23 cm (Microagar) to 3.92 ± 0.34 cm (Gelrite). Gelrite and Plant agar resulted in the highest shoot length values and were significantly higher than Microagar and Daishin agar (Tukey’s HSD test, *p* < 0.05), while Gelcarin and Phytoagar showed intermediate values and did not differ significantly from either group.

In *M*. × *piperita*, shoot length differed significantly among gelling agents (Tukey’s HSD, *p* < 0.05), with the longest shoots obtained on Gelcarin (8.20 ± 0.55 cm). Microagar (7.57 ± 0.60 cm), Plant agar (7.25 ± 0.57 cm), and Phytoagar (7.08 ± 0.49 cm) produced intermediate shoot lengths, whereas Daishin agar resulted in significantly shorter shoots (6.07 ± 0.52 cm).

A similar pattern was observed in *S. rebaudiana*, where shoot length varied significantly depending on the gelling agent used (Tukey’s HSD, *p* < 0.05). Gelcarin produced the longest shoots (7.74 ± 0.64 cm), while Microagar resulted in the lowest shoot length (5.14 ± 0.54 cm).

#### 2.2.2. Rooting Response

Root development was more sensitive to the type of gelling agent than to the number of shoots, with clear differences among the tested systems (Figure 3c–e).

In *H. perforatum*, the highest number of roots per explant was recorded on Gelrite (2.29) and Gelcarin (2.10), while Microagar resulted in the lowest value (0.55). These differences in root number were statistically significant among gelling agents (Tukey’s HSD test, *p* < 0.05). Rooting percentage followed a similar pattern, reaching 95% on Gelrite, compared with only 25% on Microagar and 35% on Phytoagar. These results indicate that gelling agent selection can strongly affect rooting efficiency in *H. perforatum*.

In *M.* × *piperita*, root initiation was generally high across treatments, with root number values ranging from 6.75 (Daishin agar) to 9.75 (Gelcarin). Differences in root number among gelling agents were statistically significant (Tukey’s HSD test, *p* < 0.05). The rooting percentage was 100% for all gelling agents except Microagar (75%). Root length values ranged from 1.31 ± 0.03 cm (Microagar) to 2.08 ± 0.12 cm (Phytoagar), with significant differences in root elongation observed among gelling agents (*p* < 0.05), indicating that although rooting was successful across most treatments, elongation responses still differed depending on gel type.

In *S. rebaudiana*, both root number and root elongation were influenced by gelling agent type. The highest number of roots per explant was observed on Plant agar (4.90) and Gelcarin (4.45), while Microagar and Daishin agar showed lower values (2.20 each). Differences in root number were statistically significant among treatments (Tukey’s HSD test, *p* < 0.05). Root length exhibited an even clearer variation: Gelcarin promoted the longest roots (2.86 ± 0.16 cm), whereas Daishin agar resulted in the shortest roots (0.80 ± 0.05 cm). Root length differences were statistically significant (*p* < 0.05). The rooting percentage remained high (≥90%) for most gelling agents, except for Microagar, in which rooting decreased to 75%.

These statistical differences are reflected in the grouping letters shown in Figure 3e.

#### 2.2.3. Shoot Water Content

Shoot water content (WC) showed relatively high values across treatments and species, but distinct patterns could still be observed depending on the gelling agent used (Figure 3f). In *H. perforatum*, WC ranged from 74% (Gelcarin) to 80% (Plant agar and Phytoagar), indicating a moderate variation in tissue hydration among gelling agents. In *M.* × *piperita*, WC remained consistently high (89–90%) for all gelling agents, suggesting a stable hydration status under the applied culture conditions, irrespective of gel type. In *S. rebaudiana*, WC values ranged from 86% (Daishin agar) to 90% (Plant agar and Gelrite), with intermediate values observed for Gelcarin (89%), Microagar (87%), and Phytoagar (87%), indicating limited but detectable differences in tissue water content depending on the gelling matrix. These statistical differences are reflected in the grouping letters shown in Figure 3f.

#### 2.2.4. Correlation Analysis Between Rheological Properties and In Vitro Responses

Spearman correlation analysis was performed to assess the strength and direction of relationships between rheological parameters of the culture media and in vitro responses, calculated separately for each species. The shear rate of 9.72 s^−1^ was selected as representative of the low-shear conditions relevant for static in vitro culture and was therefore used for comparative analysis and correlation with biological responses. The resulting correlation coefficients are summarized in Table 1. Full species-specific Spearman correlation matrices are provided in Supplementary Tables S1–S3 for completeness.

## 3. Discussion

While previous studies have evaluated individual gelling agents or focused primarily on biological responses, the present study provides an integrated assessment linking the rheological flow behavior of multiple commercially available gelling agents with in vitro culture performance across three medicinal plant species. In particular, the relevance of low-shear apparent viscosity as a practical selection criterion is highlighted as a useful contribution to the optimization of gel-based culture media.

### 3.1. Rheological Behavior of Gelling Agents

All six gelling agents showed pronounced shear-thinning (pseudoplastic) behavior, characterized by a non-linear decrease in apparent viscosity with increasing shear rate. This behavior is consistent with the progressive alignment and partial breakdown of the polymeric network under shear, leading to reduced flow resistance [35]. Similar shear-dependent viscosity profiles are commonly reported for polysaccharide-based gel systems and are generally associated with the disruption of intermolecular interactions and network rearrangement during deformation [36,37,38].

Overall, the rheological differences observed between the gelling agents, especially in the low-shear regime, suggest differences in gel network strength and mechanical stability. These variations may be relevant for in vitro culture applications, where the gel matrix contributes to explant support and water availability, and were therefore further evaluated through plant growth responses in the subsequent section.

### 3.2. Integrated Interpretation of Rheological and In Vitro Responses

Regarding shoot induction and elongation, our results indicate that gelling agent type had a limited influence on shoot induction, whereas its effect was more evident for subsequent shoot growth, as also reported in other in vitro systems comparing agar- and gellan gum-based gelling agents [34]. Buah et al. [39] reported that in vitro banana growth was influenced by both the type and concentration of gelling agent, mainly due to differences in the physical properties of the culture medium. The authors also emphasized that each gelling agent may have an optimal concentration, and that Gelrite promoted superior growth and multiplication under their experimental conditions. Similarly, Gelrite has been reported as an effective alternative to agar for in vitro propagation of oil palm [40]. Therefore, gelling agents can influence shoot elongation under PGR-free conditions, potentially through differences in gel structure, mechanical support, and diffusion-related properties of gelled media, as previously highlighted for tissue culture systems [12,41,42,43].

The physical constraints imposed by the gel matrix also influenced root development. High concentrations of agar have been found to reduce in vitro growth and rooting due to increased mechanical restrictions and reduced nutrient availability within the culture medium [2,44]. Root elongation may be particularly sensitive to the physical properties of the gel medium, potentially reflecting differences in mechanical resistance and water mobility within the gel matrix [45,46].

Gelling agents can influence tissue hydration and water availability, although the effect appears to be species-dependent. The influence of gelling agent type and concentration on tissue hydration has also been reported previously, with agar and Gelrite inducing distinct water content and hyperhydricity responses in *Gypsophila paniculata* [47]. In this context, it has been suggested that differences in gel firmness and water retention may affect moisture status in vitro and contribute to physiological disorders such as hyperhydricity, emphasizing the importance of selecting appropriate gelling agents for culture media [48]. In addition, recent work has highlighted that the physical and mechanical properties of gelled media can be linked to in vitro tissue responses and may affect water availability at the tissue–medium interface [4].

The tested systems showed pronounced shear-thinning behavior, and the major differences among gelling agents were evident at low shear rates, which are most relevant for static in vitro culture conditions. Gelling agents with lower to intermediate apparent viscosity tended to support improved shoot elongation and/or rooting performance in several cases, with Gelrite and Gelcarin frequently ranking among the top-performing treatments depending on species and parameter. At the same time, the highest-viscosity system (Daishin agar) frequently ranked among the lowest-performing treatments and was associated with reduced shoot length in *M.* × *piperita* and reduced root length in *S. rebaudiana*. These results also support the concept that excessively firm or “over-solidified” media may negatively affect in vitro performance by increasing mechanical impedance and limiting effective water and solute mobility at the explant–medium interface. In line with this, high agar concentrations have been reported to reduce in vitro growth and rooting due to mechanical restrictions and reduced nutrient availability within the culture medium [2]. Indeed, the selection of gelling agents and their specific concentrations is a critical factor in in vitro systems, often acting as a bottleneck for plant development, as recently shown in *Ipomoea batatas* [6]. In that study, as well as in our findings, the physical matrix of the gel regulates the availability and diffusion of nutrients and water to the explant, where different gel strengths directly influence root and shoot parameters. Overall, these findings support the idea that gelling agents should be selected not only based on their ability to solidify the medium but also by considering their rheological and physical properties that may affect the in vitro development of explants [12]. These results are also consistent with previous studies, which report that rooting and regeneration responses can vary depending on the type of gelling agent and culture conditions [48]. This is also supported by recent observations in *S. rebaudiana*, showing that in vitro growth outcomes may differ depending on the gelling matrix used [49].

When considered together with the rheological characterization, the in vitro results support the relevance of gel flow properties for gelling agent selection, an interpretation that is quantitatively explored through species-specific correlation analysis (Table 1). Gelling agents characterized by lower to intermediate apparent viscosity frequently ranked among the best-performing treatments for shoot elongation and rooting traits. In this context, Gelrite (5311 mPa·s) and Gelcarin (10,324 mPa·s) consistently showed favorable biological responses, whereas the highest-viscosity system, Daishin agar (30,173 mPa·s), was associated with reduced growth performance across species.

Among agar-based gelling agents, Plant agar (17,113 mPa·s) and Phytoagar (11,699 mPa·s) displayed intermediate viscosity values and showed the most favorable responses depending on species and parameter, further supporting the link between rheological properties and biological performance. This relationship is reflected in the correlation analysis, where apparent viscosity showed a strong positive correlation with rooting percentage in *S. rebaudiana* (ρ = 0.88, *p* < 0.05). In *M.* × *piperita*, increased apparent viscosity was associated with strong negative monotonic relationships with shoot elongation (ρ = −0.71) and root number (ρ = −0.77) while showing a positive relationship with rooting percentage (ρ = 0.66).

In summary, gelling agent performance was species- and trait-dependent (Table 2), supporting the relevance of gel structure and low-shear flow behavior as practical criteria for optimizing in vitro culture media. Together with the species-specific correlation analysis (Table 1), these results indicate that gel rheology contributes to differences in shoot growth and rooting responses among gelling agents.

In addition to biological performance and rheological behavior, gelling agent selection in routine micropropagation may also be influenced by economic considerations, particularly when media are prepared at a large scale. In this study, the estimated cost per liter of culture medium varied markedly among gelling agents (Table 3), ranging from 0.39 €/L for Gelrite to 1.31 €/L for Daishin agar. Similar cost-related considerations have been previously highlighted in micropropagation systems, where medium solidification represents an important practical aspect when selecting gelling agents for efficient in vitro production [50].

Despite these clear trends, several factors should be considered when interpreting the present results. This study was conducted using a basal medium formulation without plant growth regulators (PGRs), which can be considered a limitation. Therefore, it would be of interest to further investigate whether the observed relationships between gelling agent rheology and in vitro performance are maintained under PGR-supplemented conditions. In this study, only steady shear measurements were used for rheological characterization. Future studies could provide additional insights by incorporating oscillatory rheology, diffusion-related analyses, and long-term subculture experiments to better capture gel behavior under extended in vitro culture conditions.

## 4. Conclusions

This study compared the rheological flow behavior of six gelling agents (Daishin agar, Gelcarin, Gelrite, Microagar, Phytoagar, and Plant agar) and evaluated their functional performance in in vitro culture of *H. perforatum*, *M.* × *piperita*, and *S. rebaudiana*. All tested gels exhibited pronounced shear-thinning behavior, with substantial differences in apparent viscosity at low shear rates. These rheological differences were associated with species-dependent responses in shoot growth, rooting performance, and shoot water content under PGR-free culture conditions. In particular, gelling agents with lower to intermediate apparent viscosity generally supported improved shoot elongation and/or rooting responses, while the highest-viscosity system (Daishin agar) was linked to reduced shoot length in *M.* × *piperita* and reduced root elongation in *S. rebaudiana*. In addition, *H. perforatum* exhibited marked differences in rooting efficiency among gelling agents, indicating a strong dependence on gel physical properties. Overall, the results highlight the relevance of low-shear rheological properties as a practical and cost-aware criterion for selecting suitable gelling agents, providing an integrated framework that links rheological behavior, biological performance, and economic efficiency in medicinal plants in vitro culture systems.

Future studies should further evaluate the performance of gelling agents under PGR-supplemented culture conditions and extend rheological characterization using oscillatory measurements and diffusion-related analyses. Such approaches would provide a more comprehensive understanding of gel behavior and its impact on long-term in vitro culture performance.

## 5. Materials and Methods

### 5.1. Rheological Measurements

Rheological measurements were performed using an Anton Paar MCR 72 modular, compact rheometer (Anton Paar, Graz, Austria) equipped with a Peltier-controlled parallel plate system (P-PTD 200/Air). Although the system allows precise temperature regulation, all measurements were conducted at room temperature (22 ± 1 °C) to ensure consistency across samples.

Approximately 3 mL of each agar gel sample was loaded between smooth parallel plates with a diameter of 50 mm, using a fixed gap of 1 mm. Excess material was carefully removed, and samples were allowed to rest for 5 min prior to testing to achieve thermal and structural equilibrium.

Flow behavior was evaluated under steady shear conditions by applying a linearly increasing shear rate from 5 to 300 s^−1^. All measurements were performed in duplicate to ensure reproducibility [35].

### 5.2. Plant Materials and In Vitro Culture Conditions

In vitro cultures of shoots of *H. perforatum*, *M.* × *piperita*, and *S. rebaudiana* were used for this experiment. All explants were collected from plantlets at the same subculture number (fifth subculture) and of comparable size and developmental stage prior to inoculation. The three species were grown in vitro on Driver and Kuniyuki Walnut (DKW) medium [51] without plant growth regulators (PGRs) and solidified with 5 g/L (*w*/*v*) Plant agar. The DKW medium was selected based on its reported suitability for the in vitro culture of medicinal and aromatic plant species, including *H. perforatum*, *M.* × *piperita*, and *S. rebaudiana* [52]. Shoots obtained after one month of in vitro culture served as explants for testing the six gelling agents. For the experiment, in vitro cultures were established on DKW medium supplemented with 30 g/L (*w*/*v*) sucrose and without PGRs. The culture media were solidified using different gelling agents (Table 3).

The concentrations were selected based on supplier recommendations and further adjusted through preliminary laboratory optimization to ensure comparable gel firmness and adequate physical support for the explants.

The pH of the media was adjusted to 5.8 prior to the addition of the gelling agents using 1 M NaOH or 1 M HCl, as required, and measured with a calibrated pH meter (WTW inoLab, Weilheim, Germany).

Glass culture vessels (720 mL; 13.5 cm × 9 cm diameter) equipped with polypropylene screw caps were used. A volume of 100 mL of medium was dispensed into each vessel, followed by autoclaving at 120 °C for 20 min. Each vessel was inoculated with ten uninodal explants under aseptic conditions, and three vessels (replicates) were used per treatment (30 explants per treatment).

After inoculation, cultures were maintained in a growth chamber under controlled environmental conditions (22 ± 1 °C, 32.4 µmol·m^−2^·s^−1^ light intensity, 16 h photoperiod). All media components were purchased from Duchefa Biochemie BV (Haarlem, The Netherlands).

After four weeks of cultivation, the following parameters were recorded: number of shoots per explant, shoot length, number of roots per explant, root length, and rooting percentage. Shoot and root length were measured manually using a millimeter-scale ruler. Rooting percentage was calculated as (number of rooted explants/total number of explants) × 100.

Shoot water content (WC) was calculated according to the following equation [53]:

WC (%) = ((Fresh Weight − Dry Weight)/Fresh Weight) × 100

WC was determined by drying the samples in a forced-air oven at 65 °C until constant weight (48 h). Dry mass was recorded using an analytical balance with a precision of ±0.0001 g.

### 5.3. Statistical Analysis

All data are expressed as mean ± standard error (SE). Within each species, differences among gelling agents were evaluated using one-way analysis of variance (one-way ANOVA). When significant differences were detected, mean comparisons were performed using Tukey’s honestly significant difference (HSD) post hoc test. Differences were considered statistically significant at *p* < 0.05. The strength and direction of correlations were quantified using Spearman’s rank correlation coefficient (ρ). Correlations were calculated separately for each species between apparent viscosity and shear stress, measured at a shear rate of 9.72 s^−1^, and in vitro growth and rooting parameters. Statistical analyses were performed using OriginPro 2021 (OriginLab Corporation, Northampton, MA, USA).

## Figures and Tables

**Table 1:** Spearman’s rank correlation coefficients (ρ) between rheological parameters of culture media and in vitro growth and rooting traits, calculated separately for each species.

Species	Rheological Parameter	No. of Shoots	Shoot Length	No. of Roots	Root Length	Rooting %	Water Content %
*H. perforatum*	Viscosity	0.14	0.03	0.03	0.14	0.14	0.14
	Shear stress	0.14	0.03	0.03	0.14	0.14	0.14
*M.* × *piperita*	Viscosity	0.39	−0.71	0.12	0.71	0.66	−0.09
	Shear stress	0.39	−0.71	0.12	0.71	0.66	−0.09
*S. rebaudiana*	Viscosity	–	−0.03	–	–	0.88 *	−0.43
	Shear stress	–	−0.03	–	–	0.88 *	−0.43

Values represent Spearman’s rank correlation coefficients (ρ). Only correlations between rheological parameters and biological traits are shown. Significant correlations (*p* < 0.05) are indicated with an asterisk (*). Dashes (–) indicate parameters not evaluated for that species. Correlations were calculated at a shear rate of 9.72 s^−1^.

**Table 2:** Summary of the best-performing gelling agents for each species based on shoot growth and rooting traits evaluated after four weeks of in vitro culture.

Species	Best for Shoot Length	Best for Number of Roots/Explant	Best for Root Length
*H. perforatum*	Gelrite/Plant agar	Gelrite/Gelcarin	Phytoagar
*M.* × *piperita*	Gelcarin	Gelcarin	Phytoagar
*S. rebaudiana*	Gelcarin	Plant agar	Gelcarin

When more than one gelling agent is listed, treatments were not significantly different (Tukey’s HSD test, *p* < 0.05).

**Table 3:** Gelling agents used in the study, supplier product codes, working concentrations, and estimated cost per liter of culture medium.

Name	Duchefa Product No.	Type	Concentration in Medium (g/L)	Price/kg (€)	Cost per Liter of Medium (€)
Daishin agar	A1111	Agar	6	219.10	1.31
Gelcarin	G1203	Carrageenan	4	145.30	0.58
Gelrite	G1101	Gellan gum (polysaccharide)	2	196.10	0.39
Microagar	M1002	Agar	4	118.60	0.47
Phytoagar	P1003	Agar	6	107.30	0.64
Plant agar	P1001	Agar	5	92.90	0.46

All gelling agents were purchased from Duchefa Biochemie BV (Haarlem, The Netherlands). Cost per liter of culture medium was calculated based on supplier list prices and the working concentration of each gelling agent. Prices according to the supplier’s online catalogue (accessed December 2025); values are shown for comparative purposes only.

## Data Availability

The original contributions presented in the study are included in the article; further inquiries can be directed to the corresponding authors.

## Supplementary Materials

The following supporting information can be downloaded at: https://www.mdpi.com/article/10.3390/gels12020163/s1, Table S1: Full Spearman correlation matrix between rheological parameters and in vitro growth and rooting traits for *Hypericum perforatum*: Table S2: Full Spearman correlation matrix between rheological parameters and in vitro growth and rooting traits for *Mentha* × *piperita*; Table S3: Full Spearman correlation matrix between rheological parameters and in vitro growth and rooting traits for *Stevia rebaudiana*.
