# Supplementary material for: Rheological Flow Behavior of Six Gelling Agents and Their Relevance for In Vitro Culture Performance of Medicinal Plants
Source: Gels. 2026 Feb 12;12(2):163. doi: 10.3390/gels12020163 (PMC12940369; doi:10.3390/gels12020163)
Supplement: Supplementary file 1 [file gels-12-00163-s001.zip › gels-4138410-supplementary.pdf]

Supplementary Table S1. Full Spearman correlation matrix between rheological parameters and in vitro growth and rooting traits for *Hypericum perforatum*

		Visc_10 s^{-1}	Stress_10 s^{-1}	No. of shoots/ explant	Shoot length	No. of roots/ explant	Root length	Rooting percentage	Water content %
Visc_10 s^{-1}	Spearman Corr.	1	1	0.14286	0.02857	0.02857	0.14286	0.14286	0.14286
Visc_10 s^{-1}	p-value	--	--	0.78717	0.95715	0.95715	0.78717	0.78717	0.78717
Stress_10 s^{-1}	Spearman Corr.	1	1	0.14286	0.02857	0.02857	0.14286	0.14286	0.14286
Stress_10 s^{-1}	p-value	--	--	0.78717	0.95715	0.95715	0.78717	0.78717	0.78717
No. of shoots/explant	Spearman Corr.	0.14286	0.14286	1	0.77143	0.77143	-0.25714	0.88571	0.2
No. of shoots/explant	p-value	0.78717	0.78717	--	0.0724	0.0724	0.62279	0.01885	0.704
Shoot length	Spearman Corr.	0.02857	0.02857	0.77143	1	0.88571	0.31429	0.94286	0.37143
Shoot length	p-value	0.95715	0.95715	0.0724	--	0.01885	0.54409	0.0048	0.46848
No. of roots/explant	Spearman Corr.	0.02857	0.02857	0.77143	0.88571	1	0.31429	0.94286	-0.08571
No. of roots/explant	p-value	0.95715	0.95715	0.0724	0.01885	--	0.54409	0.0048	0.87174
Root length	Spearman Corr.	0.14286	0.14286	-0.25714	0.31429	0.31429	1	0.14286	-0.08571
Root length	p-value	0.78717	0.78717	0.62279	0.54409	0.54409	--	0.78717	0.87174
Rooting percentage	Spearman Corr.	0.14286	0.14286	0.88571	0.94286	0.94286	0.14286	1	0.2
Rooting percentage	p-value	0.78717	0.78717	0.01885	0.0048	0.0048	0.78717	--	0.704
Water content %	Spearman Corr.	0.14286	0.14286	0.2	0.37143	-0.08571	-0.08571	0.2	1
Water content %	p-value	0.78717	0.78717	0.704	0.46848	0.87174	0.87174	0.704	--

Values represent Spearman's rank correlation coefficients (ρ). Correlations were calculated separately for each species between rheological parameters and in vitro growth and rooting traits. Correlations were calculated at a shear rate of 9.72 s^{-1}. Significant correlations ($p < 0.05$) are indicated with an asterisk (*).
Dashes (–) indicate parameters not evaluated or invariant for that species.

Supplementary Table S2. Full Spearman correlation matrix between rheological parameters and in vitro growth and rooting traits for *Mentha × piperita*

		Visc_10 s^{-1}	Stress_10 s^{-1}	No. of shoots/ explant	Shoot length	No. of roots/ explant	Root length	Rooting percentage	Water content %
Visc_10 s^{-1}	Spearman Corr.	1	1	0.39279	-0.71429	-0.77143	0.14286	0.65465	-0.08571
Visc_10 s^{-1}	p-value	--	--	0.44111	0.11079	0.0724	0.78717	0.1583	0.87174
Stress_10 s^{-1}	Spearman Corr.	1	1	0.39279	-0.71429	-0.77143	0.14286	0.65465	-0.08571
Stress_10 s^{-1}	p-value	--	--	0.44111	0.11079	0.0724	0.78717	0.1583	0.87174
No. of shoots/explant	Spearman Corr.	0.39279	0.39279	1	-0.39279	-0.13093	-0.39279	-0.2	-0.39279
No. of shoots/explant	p-value	0.44111	0.44111	--	0.44111	0.80473	0.44111	0.704	0.44111
Shoot length	Spearman Corr.	-0.71429	-0.71429	-0.39279	1	0.94286	-0.02857	-0.13093	-0.25714
Shoot length	p-value	0.11079	0.11079	0.44111	--	0.0048	0.95715	0.80473	0.62279
No. of roots/explant	Spearman Corr.	-0.77143	-0.77143	-0.13093	0.94286	1	-0.25714	-0.39279	-0.37143
No. of roots/explant	p-value	0.0724	0.0724	0.80473	0.0048	--	0.62279	0.44111	0.46848
Root length	Spearman Corr.	0.14286	0.14286	-0.39279	-0.02857	-0.25714	1	0.65465	0.77143
Root length	p-value	0.78717	0.78717	0.44111	0.95715	0.62279	--	0.1583	0.0724
Rooting percentage	Spearman Corr.	0.65465	0.65465	-0.2	-0.13093	-0.39279	0.65465	1	0.13093
Rooting percentage	p-value	0.1583	0.1583	0.704	0.80473	0.44111	0.1583	--	0.80473
Water content %	Spearman Corr.	-0.08571	-0.08571	-0.39279	-0.25714	-0.37143	0.77143	0.13093	1
Water content %	p-value	0.87174	0.87174	0.44111	0.62279	0.46848	0.0724	0.80473	--

Values represent Spearman's rank correlation coefficients (ρ). Correlations were calculated separately for each species between rheological parameters and in vitro growth and rooting traits. Correlations were calculated at a shear rate of 9.72 s^{-1}. Significant correlations ($p < 0.05$) are indicated with an asterisk (*). Dashes (–) indicate parameters not evaluated or invariant for that species

Supplementary Table S3. Full Spearman correlation matrix between rheological parameters and in vitro growth and rooting traits for *Stevia rebaudiana*

		Visc_10 s^{-1}	Stress_10 s^{-1}	No. of shoots/ explant	Shoot length	No. of roots/ explant	Root length	Rooting percentage	Water content %
Visc_10 s^{-1}	Spearman Corr.	1	1	--	-0.02857	0.11595	-0.08571	0.88041	-0.42857
Visc_10 s^{-1}	p-value	--	--	--	0.95715	0.82685	0.87174	0.0206	0.3965
Stress_10 s^{-1}	Spearman Corr.	1	1	--	-0.02857	0.11595	-0.08571	0.88041	-0.42857
Stress_10 s^{-1}	p-value	--	--	--	0.95715	0.82685	0.87174	0.0206	0.3965
No. of shoots/explant	Spearman Corr.	--	--	--	--	--	--	--	--
No. of shoots/explant	p-value	--	--	--	--	--	--	--	--
Shoot length	Spearman Corr.	-0.02857	-0.02857	--	1	0.81168	0.71429	-0.03036	0.65714
Shoot length	p-value	0.95715	0.95715	--	--	0.04986	0.11079	0.95448	0.15617
No. of roots/explant	Spearman Corr.	0.11595	0.11595	--	0.81168	1	0.69573	0.15401	0.72471
No. of roots/explant	p-value	0.82685	0.82685	--	0.04986	--	0.12479	0.77081	0.10324
Root length	Spearman Corr.	-0.08571	-0.08571	--	0.71429	0.69573	1	-0.03036	0.31429
Root length	p-value	0.87174	0.87174	--	0.11079	0.12479	--	0.95448	0.54409
Rooting percentage	Spearman Corr.	0.88041	0.88041	--	-0.03036	0.15401	-0.03036	1	-0.27323
Rooting percentage	p-value	0.0206	0.0206	--	0.95448	0.77081	0.95448	--	0.60035
Water content %	Spearman Corr.	-0.42857	-0.42857	--	0.65714	0.72471	0.31429	-0.27323	1
Water content %	p-value	0.3965	0.3965	--	0.15617	0.10324	0.54409	0.60035	--

Values represent Spearman's rank correlation coefficients (ρ). Correlations were calculated separately for each species between rheological parameters and in vitro growth and rooting traits. Correlations were calculated at a shear rate of 9.72 s^{-1}. Significant correlations ($p < 0.05$) are indicated with an asterisk (*). Dashes (–) indicate parameters not evaluated or invariant for that species.
